# The quiet crossing of ocean tipping points

**DOI:** 10.1073/pnas.2008478118

**Published:** 2021-02-22

**Authors:** Christoph Heinze, Thorsten Blenckner, Helena Martins, Dagmara Rusiecka, Ralf Döscher, Marion Gehlen, Nicolas Gruber, Elisabeth Holland, Øystein Hov, Fortunat Joos, John Brian Robin Matthews, Rolf Rødven, Simon Wilson

**Affiliations:** ^a^Geophysical Institute, University of Bergen, 5020 Bergen, Norway;; ^b^Bjerknes Centre for Climate Research, University of Bergen, 5020 Bergen, Norway;; ^c^Stockholm Resilience Centre, Stockholm University, 10691 Stockholm, Sweden;; ^d^Rossby Centre, Swedish Meteorological and Hydrological Institute, 60176 Norrköping, Sweden;; ^e^Laboratoire des Sciences du Climat et de l’Environnement, Institut Pierre Simon Laplace, 91191 Gif-sur-Yvette cedex, France;; ^f^Institute of Biogeochemistry and Pollutant Dynamics, Eidgenössische Technische Hochschule (ETH) Zürich, 8092 Zürich, Switzerland;; ^g^Pacific Centre for the Environment and Sustainable Development, The University of the South Pacific, Suva, Fiji;; ^h^Norwegian Meteorological Institute, 0371 Oslo, Norway;; ^i^The Norwegian Academy of Science and Letters, 0271 Oslo, Norway;; ^j^Climate and Environmental Physics, Physics Institute, University of Bern, 3012 Bern, Switzerland;; ^k^Oeschger Centre for Climate Change Research, University of Bern, 3012 Bern, Switzerland;; ^l^School of Architecture, Computing and Engineering, University of East London, E16 2RD, London, United Kingdom;; ^m^Arctic Monitoring and Assessment Programme Secretariat, 9296 Tromsø, Norway

**Keywords:** ocean, biogeochemistry, climate change, tipping points, regime shifts

## Abstract

Anthropogenic climate change profoundly alters the ocean’s environmental conditions, which, in turn, impact marine ecosystems. Some of these changes are happening fast and may be difficult to reverse. The identification and monitoring of such changes, which also includes tipping points, is an ongoing and emerging research effort. Prevention of negative impacts requires mitigation efforts based on feasible research-based pathways. Climate-induced tipping points are traditionally associated with singular catastrophic events (relative to natural variations) of dramatic negative impact. High-probability high-impact ocean tipping points due to warming, ocean acidification, and deoxygenation may be more fragmented both regionally and in time but add up to global dimensions. These tipping points in combination with gradual changes need to be addressed as seriously as singular catastrophic events in order to prevent the cumulative and often compounding negative societal and Earth system impacts.

Substantial efforts have been made to carry out research and raise awareness of tipping points in the Earth system under human-induced climate change (1, 2). Singular catastrophic events (relative to natural variations) with an extraordinary (negative) global impact have received increasing attention. Some are currently assessed as having a fairly low probability of occurring before 2100. The collapse of the global ocean overturning circulation or the rapid partial disintegration of the West Antarctic Ice Sheet exemplify low-probability high-impact events with severe consequences for the Earth system. Circulation changes would cause alterations in Earth’s heat budget, while the ice sheet instability would cause a sea level rise of several meters (e.g., refs. 3 and 4). Given the considerable negative impacts were such events to occur, it is wise to minimize the associated risks through greenhouse gas (GHG) emission reductions now. Our focus here, however, is on imminent ocean changes that are already having, or will soon have, a profound impact on the marine environment, on its ecosystem goods and services, and hence on society.

The ocean is a giant reservoir of heat and dissolved carbon. Since the beginning of the industrial revolution, the oceans have taken up 30 to 40% of the total carbon dioxide (CO_2_) and 93% of the heat added to the atmosphere by human activities (5–7). The provision of this service to human societies comes with a high cost, as it causes the ocean to warm and to become more acidic, with a myriad of consequences for marine biogeochemistry and life, including the loss of oxygen (O_2_). We argue that ocean warming, ocean acidification, and ocean deoxygenation, if left unabated, have the potential to trigger a number of abrupt changes from tipping points in the marine environment, with potentially serious consequences for marine ecosystems and ocean functioning (8).

Human-caused environmental changes can materialize very rapidly, or “abruptly,” typically at rates much faster than sustained natural changes of the past (9). Such changes are already ongoing and documented for ocean warming, for acidification, and, to a certain degree, also for deoxygenation (e.g., refs. 10–14). Superimposed on fast changes in these ocean state variables are extreme events, such as heat waves, coastal hypoxia, and ocean acidification events linked, for example, to strong upwelling episodes. Since these developments are likely to aggravate over this century, they are important, as are the extraordinary abrupt singular events (e.g., refs. 2 and 15). Thus, our aim is to demonstrate that there are a number of high-probability high-impact tipping points in the ocean’s physical, chemical, and biological systems.

The term “tipping point” has a long history in chemistry and mathematics, describing a qualitative change in the characteristics of a system (a mathematical bifurcation; see ref. 16). Regime shifts (or alternative stable states) are major system reorganizations in, for example, ecosystems or socioecological systems (17, 18). Such shifts are the result of crossing a tipping point, after which the system can quickly shift away from its current state, into a contrasting, alternative state (18). The recovery to the initial system state may be difficult or even impossible. The drivers that induce a tipping point can be either a forcing or a change of the inherent system properties (such as freshwater content, bathymetry, or other system parameters) (see figure 2 in ref. 19). Here, we use the term “tipping point” according to the recent Intergovernmental Panel on Climate Change (IPCC) *Special Report on the Ocean and Cryosphere in a Changing Climate* (20): “A level of change in system properties beyond which a system reorganizes, often in a non-linear manner, and does not return to the initial state even if the drivers of the change are abated. For the climate system, the term refers to a critical threshold at which global or regional climate changes from one stable state to another stable state.”

Ref. 1 introduced the term “tipping element,” defined as a subsystem of the climate system that could experience a tipping point. Unfortunately, the phrase “tipping point” is often incorrectly used in the environmental literature, when the authors simply mean some threshold after which increasing damages or impact occur but these are not related to a mathematical bifurcation or regime shifts. In complex physical−chemical−biological marine systems, tipping points associated with nonlinear changes in these systems—relative to the forcing applied—can often not be as easily identified as in the theoretical analysis of simple dynamical systems (21). Ref. 22 (see their table 6.1) lists major marine abrupt changes and their ir-/reversibility characteristics associated with the physical and chemical forcing of the ocean resulting from anthropogenic activities. Next to abrupt changes or extreme events in circulation, stratification, and seawater temperature, changes—both gradual and abrupt—in biogeochemical variables need to be taken into account. Examples are the switch from oversaturation to undersaturation for mineral forms of calcium carbonates that make up the shells of many marine organisms (including the associated dissolution of marine seafloor calcareous sediment) or the switch from oxygenated to hypoxic or suboxic conditions. Tipping points in marine biogeochemistry and ecosystems can occur under gradual physical change, while physical changes can cascade or trigger biogeochemical and ecosystem-wide tipping points; likewise, gradual or abrupt changes in biogeochemistry can induce ecosystem-wide regime shifts (Fig. 1). For a complete risk and impact assessment concerning human-induced changes of the ocean and for mitigation strategies, both tipping points from smooth transitions of a threshold and those cascading from other tipping points, irreversibility, and abrupt changes need to be taken into account.

**Fig. 1.**
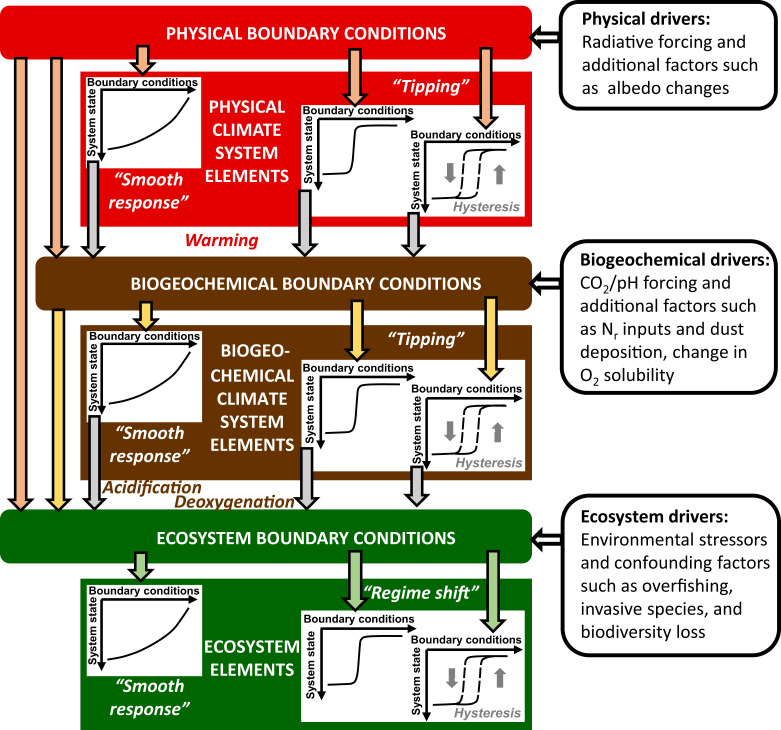
Abrupt system changes in the physical, biogeochemical, and ecosystem compartments of the ocean (big colored boxes) can be induced by external drivers (rounded black and white boxes) or by couplings between the compartments. Tipping behavior can be potentially induced by any type of boundary conditions and can cascade from a change of the system state in one compartment to the boundary conditions for another compartment. “System state” on the *y* axes of the small diagrams denotes all relevant state variables such as temperature, O_2_ concentration, pH etc. “Boundary conditions” on the *x* axes includes all relevant forcings either from external sources or from other compartments of the ocean system. N_r_ stands for reactive nitrogen (such as nutrient inputs from land). Both boundary conditions and system states are time dependent. Abrupt changes can potentially be reversible, so that one system state occurs for a unique type of conditions directly following the forcing. Particularly critical are abrupt system changes that show hysteresis. In this case, a certain system state is not coupled to one unique forcing. Strong negative forcing may be needed to enable a return to the initial system state (or such a return may be completely impossible in case of irreversibility).

We will present here 1) the evidence for high-probability high-impact tipping points in ocean physics, chemistry, and biology; 2) the challenge of dealing with the enormous spectrum of time scales governing changes in the Earth system; and 3) options for mitigation measures to avoid future tipping points.

## Ongoing Ocean Change and Tipping Points

Tipping points can be reached, for example, for certain degrees in warming, a certain decline of marine dissolved O_2_, or a certain change in acid−base chemistry through CO_2_ uptake from the atmosphere.

### Warming.

Each species of marine organisms has an optimal temperature window (niche) for its physiological functioning (23, 24). Most organisms are vulnerable to warming above this optimal temperature and are usually less vulnerable toward cooling (e.g., ref. 25). Ref. 26 concludes, with high confidence, that the majority of tropical coral reefs that exist today will disappear even if global warming is limited to 1.5 °C. These coral reef systems play an important role for fisheries, for coastal protection, as fish nurseries, and for a number of other ecosystem services (27). The effect of ocean warming extends far beyond the most sensitive marine organisms, with range shifts being observed across the food web from phytoplankton to marine mammals (28). Global fisheries catches are also expected to decline in proportion to climate warming (29, 30). As in the past, there is also the potential for unexpected climate-related regime shifts in marine ecosystems, like those observed in the North Pacific (31).

### Deoxygenation.

Most marine organisms can only exist in seawater with sufficiently high concentrations of dissolved O_2_ (32). Warming of the ocean decreases the solubility of O_2_ in seawater. Further, warming induces an acceleration of metabolic rates and thus also of O_2_ consumption (33). Further, a slowing down of ocean mixing under warming transports less O_2_ from the surface into the ocean interior, changing the balance between O_2_ supply and consumption. In addition, delivery of land-based nutrients through run-off (e.g., agricultural fertilizers, domestic waste) and deposition from the atmosphere increases biological productivity in coastal areas, disrupting ecosystems and enhancing the risk of coastal hypoxia (34). These factors cause the O_2_ drawdown in the ocean (35) with potential for large consequences in combination with warming for marine organisms (36), whose species distribution, growth, survival, and ability to reproduce are negatively affected. Current O_2_ minimum zones [with a dissolved O_2_ concentration lower than 80 µmol⋅L^−1^, which is close to the threshold of 60 µmol⋅L^−1^ below which waters become “dead zones” for many higher animals (37)] are expected to extend under climatic change if GHG emissions rise unabated (28, 38, 39). Ocean deoxygenation is sensitive to the magnitude of radiative forcing by GHGs and other agents and can persist for centuries to millennia (10, 40), although, regionally, trends can be reversed. Transiently, the global mean ocean O_2_ concentration is projected to decrease by a few percent under low forcing to up to 40% under high forcing (28), with deoxygenation peaking about a thousand years after stabilization of radiative forcing (10). Hypoxic waters will expand over the next millennium, and recovery will be slow and remains incomplete under high forcing, especially in the thermocline (41). Mitigation measures are projected to reduce peak decreases in oceanic O_2_ inventory by 4.4% per degree Celsius of avoided equilibrium warming (10).

### Ocean Acidification.

In addition to being a radiative forcing agent, CO_2_ also forces the ocean chemically: CO_2_ enters the ocean via air−sea gas exchange, and acid−base reactions between CO_2_ and seawater cause the concentration of H^+^ to increase and that of CO_3_^2-^ to decrease (42). This leads to a decrease in the saturation of seawater with respect to the mineral calcium carbonate (CaCO_3_); that is, CaCO_3_ tends to dissolve once the acidified seawater crosses the boundary between oversaturation and undersaturation. This threshold differs for the various polymorphs of CaCO_3_, that is, calcite, aragonite or high-magnesium calcite. Many marine organisms have shells or skeletal structures made of these mineral forms of CaCO_3_ and are potentially particularly vulnerable to ocean acidification. A well-known example is pteropods, aragonite-forming pelagic sea snails that are a keystone species in the marine food web (43, 44). The changes to the marine carbonate system that have occurred since the industrial revolution are already unprecedented within the last 65 million years (26). Ocean acidification conditions will prevail (and aggravate) for many centuries in the ocean interior after reduction of carbon emissions to net zero. For example, the volume of water supersaturated with respect to aragonite is progressively reduced by more than a factor of two even in scenarios where global temperature change is limited to well below 2 °C (45). The detection of ocean tipping points related to ocean acidification is difficult, due to the complexity of the ecosystem response (46) and the lack of long time series for monitoring of acidification-induced ecosystem responses. That Arctic Ocean surface waters may become undersaturated for aragonite seasonally and regionally from year 2016 on has been derived from Earth system modeling (14). Observations show increasing acidification in subsurface waters with aragonite undersaturation in the western Arctic Ocean (47). Fast changes in ocean acidity have also been reported and projected for the Californian upwelling system (13, 48) and the Southern Ocean (49, 50).

**Fig. 2.**
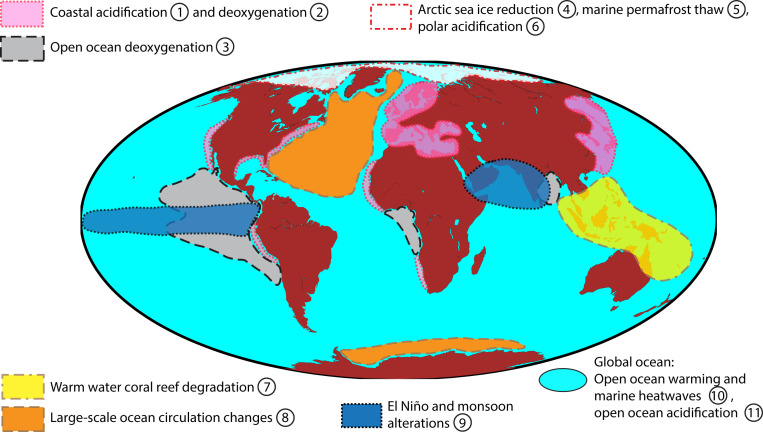
Candidates for high-probability high-impact marine tipping elements that concern warming, deoxygenation, and ocean acidification as well as their impacts. Further details concerning drivers, variables affected, and potential hazards are listed in *SI Appendix*, Table S1. The areas indicated in the map are approximate only. References/examples: ①, refs. 48 and 122; ②, refs. 11 and 34; ③, refs. 37, 123, and 124; ④, refs. 94 and 125; ⑤, ref. 62; ⑥, refs. 14 and 126; ⑦, refs. 63 and 127; ⑧, refs. 128–130; ⑨, refs. 29, 64, and 65; ⑩, refs. 12, 131, and 132; ⑪, refs. 59, 133, and 134.

Cumulative effects of warming, O_2_ loss, and pH decline may impact, synergistically, marine biota and may, in some cases, lead to ecosystem regime shifts (51). Marine regime shifts are often caused by multiple factors (52), through either climate-induced changes in multiple variables or other confounding factors such as overfishing, high human-made nutrient input from land, and invasive species (17). In addition, extreme “ocean weather” events, such as marine heatwaves (53–55) or high acidity/low saturation state events (56), can have severe consequences for marine biodiversity (57). Aggregated over the globe, the observed local and regional changes and regime shifts already add up to a substantial global problem that needs to be recognized (Fig. 2). Besides the sea surface, where warming and acidification are strongest, also the intermediate waters, especially for the eastern tropical Pacific Oxygen Minimum Zone (e.g., ref. 58), and deep-water domains such as the deep North Atlantic Ocean (59), are changing. This is of importance as deep-sea ecosystems are adapted to stable conditions, and even small changes in these conditions can have disproportionate impacts.

Candidates for high-probability high-impact ocean tipping points and tipping elements cover several regional domains (Fig. 2 and *SI Appendix*, Table S1 and references therein). Coastal acidification and deoxygenation are often linked to upwelling areas, where nutrient availability and biological production rates are high. Open ocean deoxygenation is particularly pronounced in the North Pacific, due to large-scale upwelling motion and progressive accumulation of O_2_ loss due to remineralization of organic matter. The Arctic Ocean is subject to a number of hazards—due to progressive reductions in sea ice, drastic pH lowering because of the high solubility and low CO_2_ buffering in cold waters, and the thawing of subsea permafrost areas (60–62). Tropical coral reefs are undergoing degradation/bleaching due to warming and acidification (63). Changes in global circulation, including in the strength of deep convection and upwelling, are expected under further warming, with a trend toward more sluggish conditions affecting ocean biogeochemistry, marine CO_2_ uptake, and ecosystems. Changes in key modes of climate variability (such as El Niño−Southern Oscillation) and seasonal climate patterns (Asian Monsoon) also can be abrupt and persistent and can impact marine ecosystems (29, 64, 65). In combination, these tipping elements and the associated tipping points add up to a global issue that warrants urgent attention and action.

## Trapped by Oceanic Time Scales

The upper ocean (upper few hundred meters) mixes on a timescale of decades, while the deep ocean water masses are renewed from the surface on a much longer timescale (100 y to 1,000 y). The deep ocean can be altered by climate change irreversibly for thousands of years, with the extent of the alteration dependent on the total change in climatic forcing over time.

**Fig. 3.**
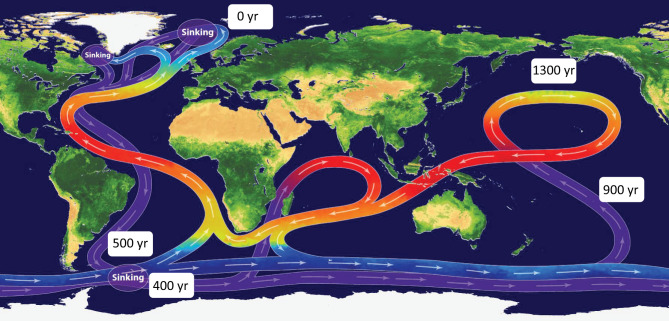
Time scale of the large-scale ocean mixing and circulation. Image credit: V. Byfield, National Oceanography Centre (NOC), licensed under CC BY 3.0. The original figure was modified with mean age indications of water masses since their last contact with atmosphere; the age indications are based on ideal age tracers following refs. 135 and 136).

The ocean is the major heat and net carbon uptake reservoir (“sink”) (6, 66, 67). Without ocean uptake, atmospheric warming would already be much larger. The present-day anomalies (relative to the preindustrial) of heat and anthropogenic carbon (and hence pH) are initially largest at the ocean surface. Through mixing and ocean currents, the anomalies for heat and carbon are transported away from the surface and enter deeper layers, from intermediate water layers down to deep and bottom waters. The amelioration of climate change is paid for by consequent changes in seawater properties and the resulting adverse effects on marine ecosystems. Apart from the decreasing solubility of O_2_ with temperature, changes in circulation alter both the supply of O_2_ to the deep ocean and the consumption of O_2_ due to organic matter degradation. The timescales of the global ocean circulation from the surface throughout the deep waters (Fig. 3) have two consequences: 1) The vertical mixing cannot prevent a strong accumulation of heat and carbon (accompanied by a pH reduction) in the upper ocean (above ∼1,000 m) if the forcing for heat and carbon is faster than the mixing of upper ocean and deep ocean. 2) Deep mixing mainly at high-latitude convective sites transports parts of the surface anomalies to greater depths, where long-lasting departures from natural values gradually build up over large volumes of ocean water. Strong hysteresis behavior with a delayed recovery of environmental variables, such as temperature or O_2_, is even projected under strong, hypothetical carbon dioxide removal from the atmosphere (68, 69).

For mitigation of climate change, these timescale aspects are important for GHG emission reductions: 1) The more the release of CO_2_ and other forcing agents to the atmosphere slows, the more efficiently excess heat and excess carbon can be transported into the deep sea, and the smaller the buildup of heat and carbon anomalies at the sea surface, acting to prevent critical tipping points in the surface and upper ocean. 2) The smaller the total amount of emitted CO_2_ becomes, the smaller the accumulation of heat and carbon in the deep sea will be, and the smaller the risk for high-magnitude irreversible change (as compared to human time scales) of the entire ocean will become.

## Mitigation Pathways to Avoid Ocean Tipping Points

The adverse impacts of human-induced climate change on the ocean can still be minimized. “Earth system targets,” like global warming or ocean acidification levels, and corresponding emission reduction targets need to be identified, agreed on, implemented, and verified. Here we highlight six areas/research topics where Earth system scientists are contributing to the development and implementation of mitigation actions.

### Identifying the Hot Spots of High-Probability High-Impact Tipping Points in the Ocean and Understanding Their Dynamics.

Scientists are employing Earth observations and a hierarchy of models, from high-resolution regional models to global models of the ocean and Earth system (e.g., ref. 70), to determine the areas where the most severe hazards have occurred in the past (decades to millions of years), are currently occurring, and may occur in the future. Advanced statistical tools are used for analyzing the datasets for signal detection of gradual and abrupt changes, for attribution to specific forcing factors, and for dynamically interpolating between observations for assessing the changing state of the ocean (71, 72). Vulnerabilities of organisms and ecosystems are studied through laboratory and in situ experiments. The probability that temporary extreme events with severe impacts will occur in the future is explored by analyzing, among others, climate projection ensembles of Earth system model simulations. Special consideration is given to compound events, where conditions are extreme in multiple parameters (73, 74), and hybrid events, where the extreme impact arises from a combination of several parameters that, in themselves, are not extreme (75). Identifying hotspots of high-impact tipping points in huge datasets may, at times, resemble finding needles in haystacks. A respective operational monitoring requires an intensive flow of high-quality data of diagnostics of the key mechanisms of the coupled Earth system. The data required are preferably provided in near real time to allow user interaction for data quality improvements (76). Another goal would be to identify important forecast failures and the reasons for them (e.g., ref. 77) through an approach following the “discovery−invention cycle” advocated by ref. 78. Current activities that point in this direction are, for example, the Seamless Global Data Processing and Forecasting System development in ref. 79 (see Goal 2 on their p. 18), and the Copernicus Program by the European Union (see https://www.copernicus.eu/en/about-copernicus, last accessed 6 November 2020). Operational prediction of ocean biogeochemical variables and fluxes in addition to physical variables on periods up to decades has made some progress recently (80, 81) but is, overall, still in its infancy.

### Assessing Ecological Tipping Points Caused by Changes in Physical and Biogeochemical Ocean Variables.

Determining definite and context-dependent thresholds for ecosystem stressors and to avoid tipping points is a difficult task due to the complexity of ecosystems, species-dependent tolerance levels and adaptive capacities, and interactive effects (e.g., refs. 51 and 82). Progress is being made to define thresholds for changes in temperature, O_2_ concentration, nutrient levels, and acidity with respect to the physiological tolerance of different key organisms (e.g., refs. 46, 61, 83, and 84). The interaction between these stressors in shaping an organism’s response to climate change is increasingly acknowledged and understood (51, 85). In recent years, we have also seen the recognition of the importance of natural short-term variability of environmental stressors, as well as the reoccurrence of extreme events [“memory effect” of recurrent heat waves for warm water coral ecosystems (86)]. Finally, understanding is growing about confounding factors inside and outside the climate change realm (such as overfishing; see, e.g., refs. 87 and 88). The respective planetary boundaries give guardrails for particularly critical issues that need to be addressed under global change (89–91).

### Identifying Multiple Mitigation Targets to Avoid Ocean Tipping Points and to Remain in Safe Operating Space.

The main environmental metrics employed to date in relation to climate change mitigation are atmospheric CO_2_ concentrations and global mean surface temperature. The simplicity of such metrics has contributed to the basis for the “Paris Agreement” (92). However, in order to avoid critical tipping points and stay within a safe operating space, more refined emission mitigation limits need to be developed and applied that address a combination of environmentally and socioeconomically relevant targets in the Earth System (93). An example is the work of the Arctic Monitoring and Assessment Program on its Arctic Climate Impact Assessment (94) and Snow, Water, Ice and Permafrost assessments (60, 95) that have highlighted the importance of the Arctic region in this respect, including the role of teleconnections. Arctic temperatures are increasing at twice the global average, with associated implications for Arctic sea ice extent/thickness, melting of ice sheets and glaciers, thawing of permafrost, etc., with impacts for Arctic ecosystems and human societies. The need to address changes in marine temperature, acidification, O_2_, and marine productivity jointly has been recognized (28, 93, 96), but multiple Earth System targets for mitigation need to be further developed and detailed.

### Assessing Different GHG Emission Scenarios (Extent, Timing) to Meet the Mitigation Targets.

Earth system models are tools to test and optimize different emission pathways toward mitigation targets (e.g., refs. 97 and 98) taking oceanic mixing timescales and carbon cycle feedbacks into account. Integrated assessment models and simple climate models (e.g., refs. 99 and 100) are used to evaluate emission reduction pathways with respect to societal feasibility and to provide emission scenarios. These are then employed in projections of Earth system models to evaluate how, for example, hazards can be reduced. The natural climate variability is an important aspect to account for to distinguish the impact of the different scenarios (101, 102).

### Evaluating Carbon Dioxide Removal, Solar Radiation Management, and Ocean Mitigation Options.

Besides the reduction of GHG emissions, a series of methods and technologies are being discussed that aim to deliberately alter the climate system (103). Their utility, feasibility, risks, as well as governance, and ethical issues are being examined. Carbon dioxide removal methods, where CO_2_ is removed from the atmosphere or ocean and stored, are generally associated with lower risks and complications than solar radiation management methods. A number of marine technical mitigation options are under discussion, also with respect to their efficiency and potential risks as well as negative side effects (104, 105). These options include mechanisms for enhancing ocean CO_2_ uptake (or preventing CO_2_ loss), such as purposeful fertilization of the ocean through iron additions (106, 107), mixing enhancement (108, 109), alkalinization (110, 111), and protection of coastal seagrass meadows and mangrove forests (112). On the other hand, methods for renewable energy production from the ocean are being developed next to the already well-established use of offshore wind energy. These energy options are based, for example, on tidal and wave energy (113) or on floating devices for solar panels and associated production of synthetic fuel (114).

### Communicating to Stakeholders.

The interaction and communication between scientists and stakeholders need improvements to increase climate and ocean literacy, and to build trust in science in societal transformation processes (22, 104). Of particular importance in this respect is improving interaction and communication with indigenous stakeholders, who not only have information (traditional and local knowledge) that can supplement science, but also play an important role in policy fora. Research on how to empower science communication to live up to its full potential and address global challenges is ongoing (see ref. 115). When scientific terminology and stakeholder terminology are overlapping, proper calibration of the different meanings needs to be ensured, as in the use of the term “uncertainty.” Societal concerns and stakeholder pressure to address specific environmental threats often shape the need for scientific assessments, as, for example, for the Minamata Convention on mercury (116).

Many of the same strategies for developing improved information for ocean mitigation approaches will be useful for adaptation approaches. Managing abrupt ocean change through mitigation measures is more effective than handling its symptoms through adaptation (117). Nevertheless, adaptation measures need to be considered in addition, in case mitigation cannot be implemented to the desired degree or quickly enough and because human-induced change of the ocean will go on for some time even if anthropogenic forcing would stop immediately. Adaptation can be structured into 1) measures that support biological and ecological adaptation [such as pollution reduction and conservation; summarize these options under the categories “protection” and “repair”] (118) and 2) measures that enhance societal adaptation (such as infrastructure based adaptation) (104).

Next, we highlight four promising options in Earth system management and societal transformation for minimizing the likelihood of encountering high-probability high-impact ocean tipping points.

1) GHG emission reductions need to be realized: The highest priority for ocean damage limitation is the immediate and drastic reduction of GHG emissions, in particular, CO_2_ emissions, since they drive ocean acidification as well as global warming and, as a consequence, deoxygenation. The smaller the excess carbon and heat uptake from human activities will be, the smaller the long-lasting changes in ocean properties and the smaller the number of regime shifts.
2) A sound global resource management needs to be implemented: To achieve emission reductions, human societies need to tackle the transformation to decarbonized energy production, sustainable use of land and ocean (including food production), and climate-friendly urban as well as regional planning in order to avoid problematic path dependencies and lock-in situations. Confounding ecosystem stressors such as overfishing, contamination with plastics, organic pollutants as well as other hazardous substances, and eutrophication need to be addressed in parallel to CO_2_ emission reductions to avoid biodiversity loss and ecosystem regime shifts.
3) The implementation of mitigation measures needs to be enabled through adequate governance structures and seamless interagency action: Mitigation measures must build on adequate implementation mechanisms. Agencies ranging from intergovernmental organizations to societal sector ministries and local authorities and networks need to work hand in hand with clear responsibilities and obligations (22). Public and private stakeholders need to synchronize appropriate actions [see also Sendai Framework (119)]. Communities need to be empowered with the capacities for transformation. Ocean monitoring must be further developed to assess compliance with agreed mitigation measures.
4) Transformations need to be carried out increasingly fast: The support for significant societal transformations to avoid triggering harmful Earth system tipping points is progressing (120). For example, the European Union has the goal to become carbon neutral by year 2050 (121).

Climate change in the ocean is manifesting itself clearly now. The cross-chapter assessment of (potentially) abrupt and irreversible phenomena related to the ocean and the cryosphere published by the IPCC in the *Special Report on the Ocean and Cryosphere in a Changing Climate* (see table 6.1 in ref. 22) lists ocean deoxygenation and ocean acidification as irreversible for centuries to millennia at depth. Abrupt physical ocean changes due to marine heatwaves are expected with very high likelihood and high confidence concerning negative impacts on ecosystems. Increased heatwave occurrences are not reversible on short time scales and would persist from decades to centuries. The physical−chemical−biological ocean systems are at the verge of tipping into another state in many oceanic regions. Integrated over the world ocean, this adds up to a global issue of concern.

## Supplementary Material

Supplementary File

## Data Availability

There are no data underlying this work.
