# Supplementary material for: The quiet crossing of ocean tipping points
Source: Proc Natl Acad Sci U S A. 2021 Feb 22;118(9):e2008478118. doi: 10.1073/pnas.2008478118 (PMC7936299; doi:10.1073/pnas.2008478118)
Supplement: Supplementary File [file pnas.2008478118.sapp.pdf]

**SI Appendix. Supporting Information.**

**Table S1.** Candidates for high-probability high-impact tipping points and tipping elements.

| **Area/tipping element** | **Driver** | **Physical-biogeochemical variables affected** | **Hazards concerning:** | **Referen-ces and examples** |
|---|---|---|---|---|
| Coastal acidification | $C_{anthro}$, $\mathbf{v_{ocean}}$ | DIC, pH, $\Omega_{calcite}$, $\Omega_{aragonite}$ | Coastal ecosystem functioning, key species, marine living resources | (1, 2) |
| Coastal deoxygenation | ΔT, $\mathbf{v_{ocean}}$ | $O_2$ | Coastal ecosystem functioning, key species, marine living resources, further eutrophication, harmful algae blooms | (3, 4) |
| Open ocean deoxygenation | ΔT, $\mathbf{v_{ocean}}$ | $O_2$ | Marine living resources | (5-7) |
| Arctic sea ice reduction | ΔT, $\mathbf{v_{ocean}}$ | Light availability, biological primary production, T | Shift in ecosystem intensification away from coast, warming, invasive species, species loss, marine living resources | (8, 9) |
| Marine permafrost thaw | ΔT, $\mathbf{v_{ocean}}$ | DIC, pH, $\Omega_{calcite}$, $\Omega_{aragonite,}$ | $CH_4$ outgassing, increase in ocean dissolved carbon, less ocean $CO_2$ uptake, atmospheric $CO_2$ | (10) |
| Polar acidification | $C_{anthro}$ | DIC, pH, $\Omega_{calcite}$, $\Omega_{aragonite}$ | Ecosystem functioning, key species, marine living resources | (11, 12) |
| Warm water coral reef degradation | ΔT, $C_{anthro}$ | pH, T | Coral bleaching, ecosystem functioning, species loss, coastal erosion, key species, marine living resources | (13, 14) |
| Large-scale ocean circulation changes (deep convection, upwelling, overturning) | ΔT, ΔS | DIC, Alk, pH, $\Omega_{calcite}$, $\Omega_{aragonite}$, $O_2$, T, S, nutrients, biological primary production | Ecosystem functioning, ocean $CO_2$ uptake, heat accumulation, pH change accumulation | (15-17) |
| El Niño and Monsoon alterations | ΔT, ΔS, $\mathbf{v_{ocean}}$ | DIC, Alk, pH, $\Omega_{calcite}$, $\Omega_{aragonite}$, $O_2$, T, S, nutrients, biological primary production | Ecosystem functioning, marine living resources, ocean $CO_2$ uptake, heat accumulation, accumulation of pH changes | (18-20) |
| Open ocean warming and marine heat waves | ΔT | T, biological primary production | Ecosystem functioning, marine living resources, ocean $CO_2$ uptake, heat accumulation | (21-23) |
| Open Ocean acidification | $C_{anthro}$, $\mathbf{v_{ocean}}$ | DIC, pH, $\Omega_{calcite}$, $\Omega_{aragonite}$ | Ecosystem functioning, key species, marine living resources, deep-sea ecosystems | (24-26) |

**See following pages for explanation of symbols and references.**

**Explanation of symbols:**
$\mathbf{v}_{ocean}$: ocean velocity field
$C_{anthro}$: additional ocean carbon from uptake of atmospheric excess $CO_2$
T: seawater temperature
ΔT: temperature change
S: salinity
ΔS: salinity change due to change in fresh water fluxes including ice melt
DIC: total dissolved inorganic carbon
Alk: alkalinity (ability of sea water to dissociate $CO_2$)
pH: ph-value, $pH=-log_{10}([H^+])$.
$\Omega_{calcite}$: saturation state for calcite ($CaCO_3$), the boundary between over- and undersaturation is at $\Omega_{calcite}=1$
$\Omega_{aragonite}$: saturation state for aragonite ($CaCO_3$), the boundary between over- and undersaturation is at $\Omega_{aragonite}=1$
$O_2$: dissolved oxygen

**SI references list:**

1. R. A. Feely, C. L. Sabine, J. M. Hernandez-Ayon, D. Ianson, B. Hales, Evidence for upwelling of corrosive "acidified" water onto the continental shelf. *Science* **320**, 1490-1492 (2008).
2. A. C. Franco, N. Gruber, T. L. Frölicher, L. K. Artman, Contrasting Impact of Future $CO_2$ Emission Scenarios on the Extent of $CaCO_3$ Mineral Undersaturation in the Humboldt Current System. *J Geophys Res-Oceans* **123**, 2018-2036 (2018).
3. F. Chan *et al.*, Emergence of anoxia in the California current large marine ecosystem. *Science* **319**, 920-920 (2008).
4. R. J. Diaz, R. Rosenberg, Spreading dead zones and consequences for marine ecosystems. *Science* **321**, 926-929 (2008).
5. R. F. Keeling, A. Körtzinger, N. Gruber, Ocean Deoxygenation in a Warming World. *Annual Review of Marine Science* **2**, 199-229 (2010).
6. K. E. Limburg, D. Breitburg, D.P. Swaney, G. Jacinto, Ocean Deoxygenation: A Primer. *One Earth,* **2**, 25-29 (2020).
7. L. Bopp *et al.*, Multiple stressors of ocean ecosystems in the 21st century: projections with CMIP5 models. *Biogeosciences* **10**, 6225-6245 (2013).
8. ACIA, *Impacts of a Warming Arctic: Arctic Climate Impact Assessment. ACIA Overview report.* (2004), pp. 140.
9. T. M. Lenton, Arctic Climate Tipping Points. *Ambio* **41**, 10-22 (2012).
10. N. Shakhova *et al.*, Current rates and mechanisms of subsea permafrost degradation in the East Siberian Arctic Shelf. *Nat Commun* **8** (2017).
11. N. R. Bates, J. T. Mathis, The Arctic Ocean marine carbon cycle: evaluation of air-sea $CO_2$ exchanges, ocean acidification impacts and potential feedbacks. *Biogeosciences* **6**, 2433-2459 (2009).
12. M. Steinacher, F. Joos, T. L. Frölicher, G. K. Plattner, S. C. Doney, Imminent ocean acidification in the Arctic projected with the NCAR global coupled carbon cycle-climate model. *Biogeosciences* **6**, 515-533 (2009).
13. O. Hoegh-Guldberg *et al.*, Coral reefs under rapid climate change and ocean acidification. *Science* **318**, 1737-1742 (2007).
14. T. P. Hughes *et al.*, Global warming and recurrent mass bleaching of corals. *Nature* **543**, 373-+ (2017).
15. H. L. Bryden, H. R. Longworth, S. A. Cunningham, Slowing of the Atlantic meridional overturning circulation at 25 degrees N. *Nature* **438**, 655-657 (2005).
16. G. A. Meehl *et al.*, "Global Climate Projections" in Climate Change 2007: The Physical Science Basis. Contribution of Working Group I to the Fourth Assessment Report of the Intergovernmental Panel on Climate Change, S. Solomon, D. Qin, M. Manning, Z. Chen, M. Marquis, K.B. Averyt, M. Tignor and H.L. Miller, Ed.

(Cambridge University Press, Cambridge, United Kingdom and New York, NY, USA, 2007), pp. 747-845.

17. W. Weijer *et al.*, Stability of the Atlantic Meridional Overturning Circulation: A Review and Synthesis. *J Geophys Res-Oceans* **124**, 5336-5375 (2019).
18. K. M. Brander, Global fish production and climate change. *P Natl Acad Sci USA* **104**, 19709-19714 (2007).
19. W. J. Cai *et al.*, Increasing frequency of extreme El Nino events due to greenhouse warming. *Nat Clim Change* **4**, 111-116 (2014).
20. Z. Lachkar, M. Levy, S. Smith, Intensification and deepening of the Arabian Sea oxygen minimum zone in response to increase in Indian monsoon wind intensity. *Biogeosciences* **15**, 159-186 (2018).
21. N. A. Bond, M. F. Cronin, H. Freeland, N. Mantua, Causes and impacts of the 2014 warm anomaly in the NE Pacific. *Geophys Res Lett* **42**, 3414-3420 (2015).
22. T. L. Frolicher, E. M. Fischer, N. Gruber, Marine heatwaves under global warming. *Nature* **560**, 360-+ (2018).
23. J. F. Piatt *et al.*, Extreme mortality and reproductive failure of common murres resulting from the northeast Pacific marine heatwave of 2014-2016. *Plos One* **15** (2020).
24. M. Gehlen *et al.*, Projected pH reductions by 2100 might put deep North Atlantic biodiversity at risk. *Biogeosciences* **11**, 6955-6967 (2014).
25. L. Kwiatkowski *et al.*, Twenty-first century ocean warming, acidification, deoxygenation, and upper-ocean nutrient and primary production decline from CMIP6 model projections. *Biogeosciences* **17**, 3439-3470 (2020).
26. J. C. Orr *et al.*, Anthropogenic ocean acidification over the twenty-first century and its impact on calcifying organisms. *Nature* **437**, 681-686 (2005).
